# Toward a methodical framework for comprehensively assessing forest multifunctionality

**DOI:** 10.1002/ece3.3488

**Published:** 2017-11-06

**Authors:** Stefan Trogisch, Andreas Schuldt, Jürgen Bauhus, Juliet A. Blum, Sabine Both, François Buscot, Nadia Castro‐Izaguirre, Douglas Chesters, Walter Durka, David Eichenberg, Alexandra Erfmeier, Markus Fischer, Christian Geißler, Markus S. Germany, Philipp Goebes, Jessica Gutknecht, Christoph Zacharias Hahn, Sylvia Haider, Werner Härdtle, Jin‐Sheng He, Andy Hector, Lydia Hönig, Yuanyuan Huang, Alexandra‐Maria Klein, Peter Kühn, Matthias Kunz, Katrin N. Leppert, Ying Li, Xiaojuan Liu, Pascal A. Niklaus, Zhiqin Pei, Katherina A. Pietsch, Ricarda Prinz, Tobias Proß, Michael Scherer‐Lorenzen, Karsten Schmidt, Thomas Scholten, Steffen Seitz, Zhengshan Song, Michael Staab, Goddert von Oheimb, Christina Weißbecker, Erik Welk, Christian Wirth, Tesfaye Wubet, Bo Yang, Xuefei Yang, Chao‐Dong Zhu, Bernhard Schmid, Keping Ma, Helge Bruelheide

**Affiliations:** ^1^ Institute of Biology/Geobotany and Botanical Garden Martin Luther University Halle‐Wittenberg Halle (Saale) Germany; ^2^ German Centre for Integrative Biodiversity Research (iDiv) Halle‐Jena‐Leipzig Leipzig Germany; ^3^ Chair of Silviculture Faculty of Environment and Natural Resources University of Freiburg Freiburg Germany; ^4^ Institute of Plant Sciences University of Bern Bern Switzerland; ^5^ Institute of Biological and Environmental Sciences University of Aberdeen Aberdeen UK; ^6^ Department of Soil Ecology Helmholtz Centre for Environmental Research – UFZ Halle (Saale) Germany; ^7^ Department of Evolutionary Biology and Environmental Studies University of Zurich Zurich Switzerland; ^8^ Institute of Zoology Chinese Academy of Sciences Beijing China; ^9^ Department of Community Ecology Helmholtz Centre for Environmental Research – UFZ Halle (Saale) Germany; ^10^ Institute of Biology University of Leipzig Leipzig Germany; ^11^ Institute for Ecosystem Research/Geobotany Kiel University Kiel Germany; ^12^ Institute of Geography, Soil Science and Geomorphology University of Tübingen Tübingen Germany; ^13^ Department of Soil, Water, and Climate University of Minnesota, Twin Cities Saint Paul MN USA; ^14^ Institute of Ecology Leuphana University of Lüneburg Lüneburg Germany; ^15^ Department of Ecology College of Urban and Environmental Sciences Key Laboratory for Earth Surface Processes of the Ministry of Education Peking University Beijing China; ^16^ Department of Plant Sciences University of Oxford Oxford UK; ^17^ Nature Conservation and Landscape Ecology Faculty of Environment and Natural Resources University of Freiburg Freiburg Germany; ^18^ Institute of General Ecology and Environmental Protection Technische Universität Dresden Tharandt Germany; ^19^ Faculty of Biology University of Freiburg Geobotany, Freiburg Germany; ^20^ Faculty of Soil and Water Conservation Beijing Forestry University Haidian District Beijing China; ^21^ State Key Laboratory of Vegetation and Environmental Change Institute of Botany Chinese Academy of Sciences Beijing China; ^22^ Senckenberg Biodiversity and Climate Research Centre (BIK‐F) Frankfurt am Main Germany; ^23^ Key Laboratory of Speciality Plant Resources of Jiangxi Province Jingdezhen University Jingdezhen China; ^24^ Kunming Institute of Botany Chinese Academy of Sciences Kunming China

**Keywords:** BEF‐China, forest biodiversity experiments, high‐throughput methods, multitrophic interactions, standardized protocols

## Abstract

Biodiversity–ecosystem functioning (BEF) research has extended its scope from communities that are short‐lived or reshape their structure annually to structurally complex forest ecosystems. The establishment of tree diversity experiments poses specific methodological challenges for assessing the multiple functions provided by forest ecosystems. In particular, methodological inconsistencies and nonstandardized protocols impede the analysis of multifunctionality within, and comparability across the increasing number of tree diversity experiments. By providing an overview on key methods currently applied in one of the largest forest biodiversity experiments, we show how methods differing in scale and simplicity can be combined to retrieve consistent data allowing novel insights into forest ecosystem functioning. Furthermore, we discuss and develop recommendations for the integration and transferability of diverse methodical approaches to present and future forest biodiversity experiments. We identified four principles that should guide basic decisions concerning method selection for tree diversity experiments and forest BEF research: (1) method selection should be directed toward maximizing data density to increase the number of measured variables in each plot. (2) Methods should cover all relevant scales of the experiment to consider scale dependencies of biodiversity effects. (3) The same variable should be evaluated with the same method across space and time for adequate larger‐scale and longer‐time data analysis and to reduce errors due to changing measurement protocols. (4) Standardized, practical and rapid methods for assessing biodiversity and ecosystem functions should be promoted to increase comparability among forest BEF experiments. We demonstrate that currently available methods provide us with a sophisticated toolbox to improve a synergistic understanding of forest multifunctionality. However, these methods require further adjustment to the specific requirements of structurally complex and long‐lived forest ecosystems. By applying methods connecting relevant scales, trophic levels, and above‐ and belowground ecosystem compartments, knowledge gain from large tree diversity experiments can be optimized.

## 1 INTRODUCTION

Biodiversity–ecosystem functioning (BEF) research requires comprehensive methodical approaches to study overall ecosystem functioning based on the simultaneous assessment of multiple functions and services. Integral approaches that include species interactions and trophic networks are especially important because ecosystem performance strongly depends on complex interactions among organisms with tight interconnections of above‐ and belowground systems (De Deyn & van der Putten, 2005; Kardol & Wardle, 2010; Soliveres et al., 2016). This is particularly true for forests, which represent long‐lived and highly complex dynamic systems (Scherer‐Lorenzen, Körner, & Schulze, 2005).

Forests support a wealth of ecosystem functions and services, such as biomass production, carbon storage, and prevention of soil erosion, and promote the diversity of coexisting taxa (Pan, Birdsey, Phillips, & Jackson, 2013). Tree diversity has been shown to affect this multifunctionality at local and larger spatial scales (Gamfeldt et al., 2013; van der Plas et al., 2016; Scherer‐Lorenzen, 2014). However, experimental research on the relationships between biodiversity and multiple ecosystem functions in forests has begun only recently (Scherer‐Lorenzen et al., 2005; Verheyen et al., 2016). Considering the complexity of forest ecosystems, it is clear that the role of tree species richness and associated diversity of microorganisms and animal taxa, including their interactions, for ecosystem functioning can only be studied adequately in a multifunctional framework (Gamfeldt, Hillebrand, & Jonsson, 2008; Hector & Bagchi, 2007).

Although observational studies along natural forest diversity gradients have offered new insights into BEF relationships, their information value is often limited by inseparable effects of species diversity and identity as well as confounding abiotic factors (Nadrowski, Wirth, & Scherer‐Lorenzen, 2010; Vilà et al., 2005). Thus, well‐designed biodiversity experiments are required to study causal tree diversity effects on ecosystem functioning and the underlying mechanisms (Hector et al., 2011; Nadrowski et al., 2010). Over the last 15 years, an increasing number of large‐scale forest diversity experiments has been established in different parts of the world, forming a growing global collaborative experimental network (www.treedivnet.ugent.be) of currently 25 tree diversity experiments (Verheyen et al., 2016). Despite their relatively young age, these planted forests already allow the evaluation of a large range of ecosystem functions also encountered in mature forests. In addition, they represent a unique large‐scale field network to study tree establishment as a function of forest diversity soon after planting and during canopy closure (Scherer‐Lorenzen, Potvin, et al., 2005).

One of the most striking features of many forest BEF experiments, in which tree species richness and composition are manipulated deliberately, is their much larger spatial dimension than comparable grassland BEF experiments. Forest BEF experiments with up to several hundred thousands of tree individuals planted often extend to the landscape scale. In small‐scale grassland BEF experiments with fast‐growing herbaceous species, environmental factors can be controlled reasonably well through applying a randomized block design. In contrast, at the landscape scale and in long‐lived tree communities, it is more difficult to ensure spatial and temporal homogeneity within the necessarily larger blocks (and plots within blocks), thus increasing the chances of accidental confounding of randomized planting with abiotic environmental variables. Thus, the separation of treatment (biodiversity) factors and environmental covariates in explaining the variation in measured ecosystem functions remains challenging in forest BEF experiments (Balvanera et al., 2006; Bruelheide et al., 2014; Caspersen & Pacala, 2001; Healy, Gotelli, & Potvin, 2008). Consequently, the methods applied to assess ecosystem functions must be applicable to capture the variation in environmental gradients and the effects of tree diversity at the different spatial scales between and within blocks (and plots). Therefore, practical, repeatable, and standardized high‐throughput methods are required to quantify ecosystem functions or variables on a large set of plots and across the network of diversity experiments. However, many currently applied BEF methods strongly differ in terms of scope and scale, complicating efficient cross‐site comparisons and synthesis approaches.

In principle, measurements of processes in forest BEF experiments typically focus on two or three spatial scales corresponding to tree community organizational levels: the individual tree, the local neighborhood of the individual tree, and the plot or community level. The level of the individual tree is used, for example, to measure species‐specific tree growth (Li, Härdtle, et al., 2014), herbivory (Schuldt, Bruelheide, et al., 2015), or fungal infestation (Hantsch, Bien, et al., 2014). Moreover, the assessment of functional plant traits is based on the measurement of individual trees with a strong focus on species identity (Kröber, Li, et al., 2015). Even if measurements are carried out on single leaves or branches, they will also refer to a particular tree individual (Brezzi, Schmid, Niklaus, & Schuldt, 2017). The local neighborhood comprises all immediate neighbor trees of a focal tree individual (Fichtner et al., 2017). Defining neighborhood in this way makes it independent of tree size. How the local neighborhood influences individual tree performance is of particular importance because positive tree–tree interactions at the local scale may translate into positive biodiversity effects at community scale (Forrester & Bauhus, 2016; Potvin & Dutilleul, 2009). In contrast, plot‐level measurements integrate ecosystem functions over the entire tree community. Such measurements are used, for example, to quantify the impact of tree species richness and composition on decomposition processes (Eichenberg et al., 2017; Seidelmann, Scherer‐Lorenzen, & Niklaus, 2016). Plot‐level measurements also apply to mobile organisms at higher trophic levels that are not confined to particular trees (Vehviläinen, Koricheva, & Ruohomäki, 2008) and to combined effects of soil fertility and topography on tree growth (Scholten et al., 2017).

Given that each method aims to contribute information at the respective scale, a well‐balanced mixture of methods is required to maximize knowledge gain from cost‐ and labor‐intensive (land rent, plot clearing, tree planting, and weeding) forest BEF experiments. Therefore, a wide spectrum of easy and sophisticated BEF measurements must be combined in a multifunctional framework to quantify ecosystem functioning on a large set of plots. Standardized methods for key ecosystem functions (Meyer, Koch, & Weisser, 2015) and rapid biodiversity assessments (Obrist & Duelli, 2010) need to be developed or adapted for forest ecosystems to promote synthesis studies across tree diversity experiments. Because these experiments are commonly used by many research teams from different disciplines and backgrounds, careful consideration of the applied methods is required to measure and analyze data jointly and effectively. Together with an integrated project data management ensuring data harmonization, data validation, and metadata quality, synthesis projects can be catalyzed in a multifunctional context (Nadrowski et al., 2013). Only if we succeed in combining the results obtained by different methods, a coherent account of forest ecosystem functioning can be achieved.

Based on an illustrative example of a forest BEF experiment (BEF‐China), we provide an overview on state‐of‐the‐art methods currently applied in one of the largest forest biodiversity experiments worldwide. Given the increasing number of tree diversity experiments and cross‐site synthesis approaches (Verheyen et al., 2016), the present work is a first attempt to develop standardized BEF methods to measure forest multifunctionality. Methods for the assessment of multiple ecosystem functions and variables are briefly described with focus on their practicability as well as their challenges that have been encountered. In a second step, we outline how methods differing in scope and complexity can be combined to retrieve consistent data allowing novel insights into forest ecosystem functioning. Finally, we discuss and develop recommendations for the integration and transferability of diverse methodological approaches across present and future forest diversity experiments.

## 2 BEF‐China as a case study of a large tree diversity experiment

**Figure 1:**
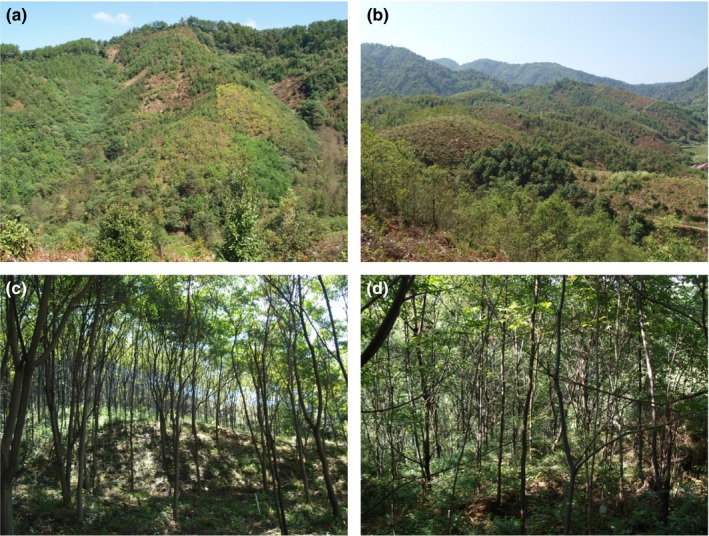
Example of a large tree diversity experiment: (a) partial view of site A and (b) site B of the BEF‐China experiment seven and six years after planting, respectively. (c) Monoculture plot of *Triadica cochinchinensis* (site A) and (d) eight‐species tree mixture of *Castanea henryi*,* Castanopsis sclerophylla*,* Choerospondias axillaris*,* Liquidambar formosana*,* Nyssa sinensis*,* Quercus serrata*,* Sapindus saponaria,* and *Triadica sebifera* (site A). To increase generality of BEF relationships, the experiment was established at two sites (about 5 km apart) with only small overlap of species pools. Photographs: S. Trogisch

BEF‐China is the first tree diversity experiment in the humid subtropics, established 2009/2010 in southeast China (Xingangshan, Jiangxi Province) with a total net area of 38.4 ha (Figure 1) distributed across two hilly landscapes (site A and B). The overall design and establishment success of the experiment are provided by Bruelheide et al. (2014) and Yang et al. (2013). A unique feature of the experiment is the large range of tree species richness levels and different nonoverlapping species combinations within different random and nonrandom (trait‐driven) extinction scenarios. The size of the total species pool is 40 tree species, and richness is varied along a log‐2 series from monocultures up to 16 species with an additional richness level of 24 species for the most diverse plots. The experiment contains more than 500 plots of 25.82 m × 25.82 m area (in horizontal projection), each planted with 400 trees in a regular grid of 20 rows × 20 columns. In two of the random extinction scenarios, tree diversity is factorially crossed with shrub diversity planted in between the trees at the same density as those. The experiment has been established on sloped terrain that allows assessing plant diversity effects on the reduction in soil erosion—an ecosystem service of high environmental importance in rain‐laden southeast China.

**Figure 2:**
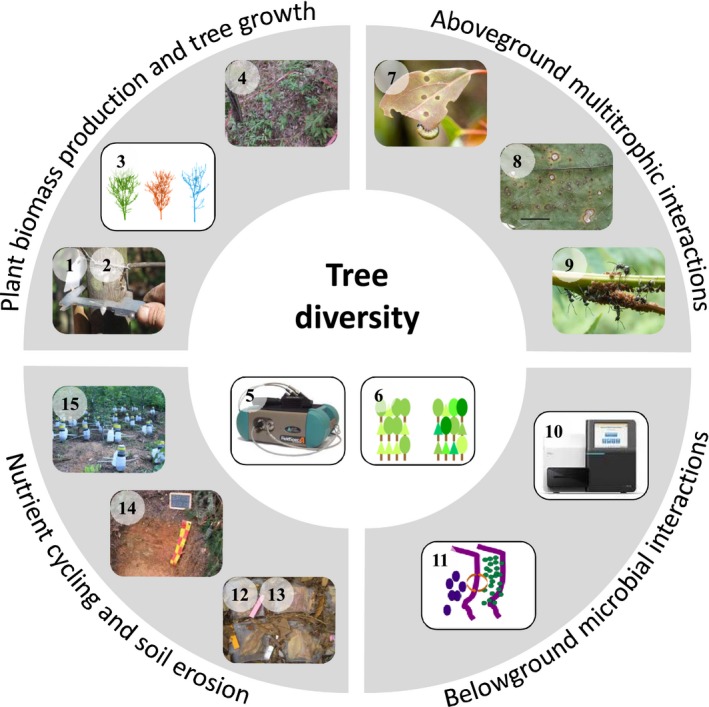
Range of methodical approaches applied in BEF‐China to study effects of tree diversity including leaf functional trait diversity (5) and genetic diversity (6) on plant biomass production and tree growth (1 + 2 = aboveground and belowground tree biomass and productivity, 3 = tree growth and canopy architecture, 4 = herb‐layer biomass and diversity), aboveground multitrophic interactions (7 = herbivory, 8 = plant‐fungal pathogens interactions, 9 = trophobiosis), belowground microbial interactions (10 = microbial diversity, 11 = microbial biomass and activity), nutrient cycling and soil erosion (12 + 13 = leaf litter and deadwood decomposition, 14 = soil fertility and C storage, 15 = soil erosion). Numbers in this figure reflect numbering of ecosystem functions and variables in Table 1

To separate tree diversity effects from influences of abiotic environmental covariates, environmental heterogeneity was quantified by assessing local and regional topography, microclimate, and edaphic conditions at the beginning of the experiment; in relation to the term landscape, we refer to this environmental heterogeneity as “ecoscape” (Bruelheide et al., 2014; Scholten et al., 2017). A wide range of functional responses and processes is being studied, such as tree growth, soil erosion, plant functional traits, importance of plant genetic diversity, plant–insect interactions, and nutrient cycling, including trophic interactions with microbial and animal decomposers. Rather than presenting an exhaustive compilation of currently obtained measurements, we provide a concise overview on key aspects of forest ecosystem functioning to illustrate the broad range of methods applied (Figure 2, Table 1). It is clear that the presented methods cannot serve as a blueprint for other tree diversity experiments but should be rather regarded as stimulus to rethink methodical concepts and approaches for large cooperative projects and networks. We begin with methods for assessing plant growth and facets of tree diversity (leaf functional trait diversity and tree genetic diversity) and extend the scope to multitrophic interactions, nutrient cycling, and soil erosion.

**Table 1:** Overview of methods for the assessment of key ecosystem functions and variables in tree diversity experiments. The spatial assessment level can be the individual tree (T), the local neighborhood (N) for studying tree–tree interactions, and the plot (P). References specific to the BEF‐China tree diversity experiment are marked with an asterisk. Temporal scope and measurement intervals for respective methods have been adapted to the requirements of BEF‐China and may depend on research focus and environmental setting

No.	Ecosystem function/variable	Method	Details/considerations	Temporal scope	Spatial assessment level (T/N/P)	References
**Plant biomass production and tree growth**						
1	Aboveground biomass and productivity	Repeated measurement of DBH (caliper, measurement tape, and dendrometer) and height (graduated pole and hypsometer)	Often only applicable for a subset of inventoried tree individuals (e.g., central 4 × 4 individuals). Allometric equations required for biomass calculation.	Annual inventory.	T	Clark, Wynne, and Schmoldt (2000) Clark et al. (2001) Li, Härdtle, et al. (2014)*
		Repeated assessment of marked leaf cohorts	Species‐specific leaf formation and longevity can be studied. Method restricted to young trees due to limited canopy access.	Half‐yearly intervals.	T	Reich, Uhl, Walters, Prugh, and Ellsworth (2004)
		Litter traps	Determination of litter production and shed leaf area. Allows quantification of nutrient fluxes from canopy to soil. Litter collection from traps on regular basis time‐consuming. Three litter traps per plot in core area (4 × 4 trees)	Biweekly litter collection over several years.	N/P	Bernier, Hanson, and Curtis (2008)
		Leaf area index (LAI)/hemispheric photography	Repeated measurements in central plot area (6 × 6 trees) allow LAI quantification during stand development. Digital hemispherical photography using a fish‐eye device less sensitive to uneven sky brightness.	Annual measurement.	N/P	Asner et al. (2003) Jonckheere et al. (2004) Peng et al. (2017)*
2	Belowground biomass and productivity	Soil cores	Destructive method for measuring root biomass, root distribution, and nutrient content. Image analyses of root scans can provide additional information on root diameter and length.	Annually or less frequently.	T/N/P	Sun et al. (2017)*
		Ingrowth cores	Destructive method for measuring root productivity.	Ingrowth core retrieval after 1 year.	T/N/P	Lei, Scherer‐Lorenzen, and Bauhus (2012) Sun et al. (2017)*
		Minirhizotrons	Nondestructive assessment of fine‐root dynamics in situ.	Pictures taken twice per year.	T/N/P	Taylor et al. (2014)
3	Tree growth and canopy architecture	Terrestrial laser scanning (TLS)	Three‐dimensional (3D) structural elements of trees. Rapid, nondestructive, accurate, and extensive measurements of a large number of individual trees over time possible.	Annually or less frequently.	T/N	Li, Hess, et al. (2014)*
4	Herb‐layer biomass and diversity	Herb‐layer monitoring	Vegetation survey by transect‐method (for inventory data). Additional composition analysis in subplot surveys. Biomass harvest in 0.5 m × 0.5 m quadrates.	Annually or less frequently.	N/P	Both et al. (2011)* Ampoorter et al. (2015) Germany et al. (2017)*
**Facets of tree diversity**						
5	Leaf functional trait diversity	Near‐infrared spectroscopy (NIRS)	Rapid and cost‐effective assessment of important leaf traits to identify linkages between functional traits and ecosystem processes. Portable NIRS allows nondestructive and highly repeated measurements in situ. Trait‐specific calibration required.	Intraday to annual measurements.	T	Serbin et al. (2014)
6	Genetic diversity	Maternal seed families, phytometer plants	Influence of seed family identity/genetic diversity on tree performance.	Annual measurements.	T	Avolio, Beaulieu, Lo, and Smith (2012) Zeng, Durka, & Fischer, (2017)* Zeng, Durka, Welk, et al. (2017)* Hahn et al. (2017)*
**Aboveground multitrophic interactions**						
7	Herbivory	Quantification of leaf damage (one‐time measurement)	Allows quick assessment of herbivory on a large number of trees. Leaf age important, thus assessment of only young and fully expanded leaves. Visually estimated leaf damage verified by leaf scans. Assessment of 6 × 6 trees in monocultures to 12 × 12 trees in more species‐rich plots.	Annually or less frequently.	T	Schuldt et al. (2012)* Schuldt, Bruelheide, et al. (2015)*
8	Plant—fungal pathogens interactions	Foliar fungal pathogens assessment	Quantification of pathogen infestation using a percentage class system of leaf damage with six damage classes. Susceptibility to pathogens as an additional species trait. Assessment of 6 × 6 trees in monocultures to 12 × 12 trees in more species‐rich plots.	Annually or less frequently.	T	Hantsch, Bien, et al. (2014)*
9	Trophobiosis	Trophobiosis as model system	Systematic survey of aphids and tending ants on at least 20 young leaves per tree. Ideal model system to quantify multitrophic interactions. Assessment of 6 × 6 trees in monocultures to 12 × 12 trees in more species‐rich plots.	Monthly survey during growing season.	T	Staab et al. (2015)*
**Belowground microbial interactions**						
10	Microbial diversity	Meta‐barcoding of rhizosphere soils using next‐generation sequencing platforms	Determine the structural and functional diversity and community composition of soil microbes (mainly fungi and bacteria). Central plot area (12 × 12 trees).	Annual measurements or less frequently.	T/N	Wu et al. (2013)* Lentendu et al. (2014)
11	Microbial biomass and activity	Phospholipid fatty acid analysis (PLFA) combined with high‐throughput method of lipid extraction; 15N dilution method, extracellular enzyme activity assays (EEA)	Determination of microbial community composition and total microbial biomass. Measurement of gross rates of N mineralization. Central plot area (12 × 12 trees).	Annual measurements or less frequently.	T/N	Oates et al. (2017) Pei et al. (2016)* Pei et al. (2017)*
**Nutrient cycling**						
12	Leaf litter decomposition	Litterbags with site‐specific or standardized leaf litter	Inexpensive, highly repeatable and time‐efficient. Standardized litter substrates (e.g., tea bags) facilitate global synthesis studies. Neglects effects of soil macrofauna.	Duration about 12 months with usually several retrieval dates.	N/P	Keuskamp et al. (2013) Trogisch et al. (2016)* Seidelmann et al. (2016)*
13	Deadwood decomposition	Litterbags with standard‐sized wood pieces	Limited to smaller wood pieces. Size of wood samples important for decomposer fauna. Easy exclusion of certain decomposers (termites) by mesh size.	Wood pieces retrieval after one and 3 years.	N/P	Russell et al. (2015) Eichenberg et al. (2017)*
14	Soil fertility and C storage	Schematic soil sampling combined with near‐infrared spectroscopy (NIRS)	Facilitate inexpensive analyses and rapid assessment of large number of samples in subsequent inventories.	Annual measurements or less frequently.	N/P	Scholten et al. (2017)* Ludwig et al. (2002)
**Soil erosion control**						
15	Throughfall kinetic energy	Splash cups	Allow indirect determination of rainfall kinetic energy at many measurement points in parallel during single rainfall events.Calibration by laser distrometer required. Eight splash cups in central plot area (6 × 6 trees).	Series of rain events.	T/N/P	Scholten et al. (2011)* Goebes, Bruelheide, et al. (2015)*
15	Soil erosion (interrill)	Microscale runoff plots	Determination of surface runoff and sediment discharge. Suitable to study vegetation effects on soil erosion processes. Five runoff plots per plot.	Series of rain events.	T/N/P	Seitz et al. (2015)* Seitz et al. (2016)*
15	Soil erosion (slope scale)	Erosion sticks	Simple and cost‐effective method to quantify large‐scale and long‐term soil erosion. Nine erosion sticks per plot.	Reading of the height above ground once per year.	N/P	Shi et al. (2011)

### 2.1 Plant biomass production and tree growth

#### 2.1.1 Aboveground tree biomass and productivity

The adequate assessment of tree biomass production in large BEF experiments is critical to investigate the influence of different facets of tree diversity (species richness, presence of particular species, species composition, functional diversity, and genetic diversity) on tree growth at the individual, neighborhood, and plot (= community) scale. Basically, tree biomass production is quantified by repeated measurements of tree size variables and subsequent calculation of tree biomass based on allometric equations, avoiding artefactual species identity effects which can be a result of using different functions for different species (e.g., Forrester, Benneter, Bouriaud, & Bauhus, 2016). However, comprehensive annual inventories with measurement of basal diameter, diameter at breast height (DBH, caliper, and measurement tape), and tree height (graduated pole for small trees and hypsometer) for all planted trees often exceed available project resources such as workforce and time. Therefore, in most cases, there is a trade‐off between the number of sampled plots and the number of sampled trees. One solution is to carry out these measurements on a section within plots. In BEF‐China, the central 16 of the 400 trees in every plot were defined as a core area and chosen for annual measurements.

In addition to quantifying woody biomass, leaf turnover has to be considered as a significant part of net primary production. Leaf production, herbivory, and mortality can be determined easily and cost‐effectively by regular monitoring of marked leaf cohorts on selected tree individuals (Brezzi et al., 2017; X. Li, unpublished data). At the beginning of the observation period, branches are marked and leaves counted. Subsequent censuses can follow at for example half‐yearly intervals, but interval length can be shorter during times of intensive growth because variable interval lengths can be accounted for using offsets in the data analysis (Egli & Schmid, 2001). Effects of tree species richness and time‐dependent covariates on leaf demographic patterns can then be estimated (Castro‐Izaguirre, 2016). Once trees have reached a certain height, community litter and seed production can be determined with litter traps (Huang et al., 2017).

The leaf area index (LAI), defined as the ratio of projected foliage area to ground area, is an important structural variable for key ecophysiological processes (e.g., energy interception and transpiration). Most commonly, LAI is indirectly measured as interception of photosynthetically active radiation (PAR) or by analysis of hemispherical photographs (Castro‐Izaguirre et al., 2016; Peng, Schmid, Haase, & Niklaus, 2017). Both methods have their advantages and disadvantages, which are further discussed in Asner, Scurlock, and Hicke (2003) and Bréda (2003).

#### 2.1.2 Belowground tree biomass and productivity

Fine roots (diameter ≤ 2 mm) are the most active part of the root system (Asaye & Zewdie, 2013), interacting with soil microflora and fauna and being involved in nutrient and water uptake (Jackson et al., 1996). Thus, understanding fine‐root dynamics is pivotal for understanding belowground interactions as well as tree growth and survival (McCormack et al., 2015). However, measuring belowground biomass and productivity is challenging as usually destructive sampling is required to separate the roots from the soil (Brassard et al., 2013). Furthermore, on sloped plots, such those in BEF‐China, an important question regarding comparability with other experiments is whether layers of soil depth should be measured perpendicular to the soil surface or to its horizontal projection. Here, our recommendation is to use a direction perpendicular to the soil surface (Sun et al., 2017).

Standing fine‐root biomass can be measured using the soil core method. Soil cores (10 cm in diameter, 30 cm in depth) are usually taken in the middle of two neighboring trees standing in the same horizontal row (Sun et al., 2017). Depending on soil type, fine roots should be sampled by soil depth increment to estimate the vertical variance of standing biomass. In BEF‐China, we were able to assign washed roots to each of the 40 species using root morphology. This allowed us to estimate the contribution of different species to overyielding of total community‐level fine‐root biomass in mixtures (Bu et al., 2017; Sun et al., 2017). In addition, roots can be scanned for analysis of diameter and specific root length (Bu et al., 2017; Sun et al., 2017). For estimation of annual production of fine roots, we recommend the traditional method of ingrowth cores (Sun et al., 2017). Right after taking the soil core for standing biomass, the cavity is refilled with sieved soil from the same plot. Ingrowth cores are resampled after 1 year, and biomass of both live and dead fine roots is measured.

As a nondestructive method, minirhizotrons have been developed to monitor fine‐root dynamics along time intervals (Guo et al., 2008; Majdi, 1996; Taylor, Beidler, Strand, & Pritchard, 2014). Minirhizotron tubes (typically length 90 cm and diameter 7 cm) are installed in the middle of two conspecific (in monoculture) or heterospecific (in mixtures) neighbored trees in an angle of 45° to the soil surface. Tubes are scanned at intervals, for example, twice per year in May and November, and pictures analyzed for fine‐root length, area, amount, longevity, and turnover rate.

#### 2.1.3 Tree growth and crown architecture

Understanding the mechanisms of biodiversity effects in forests requires information about crown structure and space partitioning between trees within and between species (Jucker, Bouriaud, Coomes, & Baltzer, 2015; Niklaus, Baruffol, He, Ma, & Schmid, 2017; Pretzsch, 2014; Schmid & Niklaus, 2017; Williams, Paquette, Cavender‐Bares, Messier, & Reich, 2017). However, conventional measurements are time‐consuming and do not deliver much detail. In recent years, terrestrial laser scanning (TLS) has been established as a time‐efficient and nondestructive approach for the measurement of the 3D structural elements of trees (Calders et al., 2015; Liang et al., 2016). Based on static laser range measurements, TLS delivers high‐resolution 3D point clouds with accuracies in the range of millimeters. In order to obtain a complete 3D picture of all focal trees in each plot, several scans from different angles are required (Watt & Donoghue, 2005). Setting up the instrument and acquiring the 3D data are generally straightforward and fast. In the case of the BEF‐China experiment, the central part of a plot with 6 × 6 trees can be captured in high detail (more than 100,000 points per tree) from nine scans within 45 min (Li, Hess, von Wehrden, Härdtle, & von Oheimb, 2014). Strong winds and occlusion by foliage may adversely affect the point cloud quality (Côté, Fournier, & Egli, 2011). Therefore, scans should to be performed under windless and—if possible—under leaf‐off conditions. Thus, in stands with deciduous trees, the preferred time for applying TLS is winter. From the resulting point clouds, a number of conventional (i.e., height and DBH), but also more complex variables (i.e., branch demography, crown volume, and wood volume), can be obtained for every tree (Kunz et al., 2017; Raumonen et al., 2013). Meanwhile, the extraction of these variables has become highly automated. However, the separation of tree individuals from a large point cloud with many trees still is a challenge, and so far, is predominantly carried out manually. With repeated TLS measurements, it is possible to quantify spatial dynamics of individual crowns and canopy filling using cylinder‐based (Raumonen et al., 2013) or voxel‐based (Hess, Bienert, Härdtle, & von Oheimb, 2015) point cloud modeling approaches.

#### 2.1.4 Herb‐layer biomass and diversity

It has been shown that trees exert strong controls on herb‐layer biomass, composition, richness, and invasibility (e.g., by altering resource availability and variability) (Ampoorter et al., 2015; Barbier, Gosselin, & Balandier, 2008; Knight, Oleksyn, Jagodzinski, Reich, & Kasprowicz, 2008; Mölder, Bernhardt‐Römermann, & Schmidt, 2008). Considering the special role of the herb layer in maintaining the structure and function of forests (Gilliam, 2007), improved understanding of how tree diversity affects herb‐layer attributes and seedling establishment is critical. In this respect, tree diversity experiments allow for assessing the relationships between forest overstory and understory species richness, composition, and productivity, and how these relationships are influenced by spatial environmental heterogeneity and forest stand age (Both et al., 2012).

In large‐scale forest experiments, full‐vegetation relevés are laborious and time‐consuming. At the plot level, W‐transects (i.e., linear transects in the shape of a W) provide a time‐ and resource‐efficient method for repetitively assessing herb‐layer species inventory as species richness and composition with information on estimated proportions in cover on a large number of plots. In addition, herb‐layer vegetation surveys performed on separate and integrated subplots (Germany, Bruelheide, & Erfmeier, 2017) can be used to explicitly test if the relationships between tree diversity and herb‐layer attributes change under variable environmental conditions (Reich et al., 2001; Weigelt, Weisser, Buchmann, & Scherer‐Lorenzen, 2009).

We recommend an integrated manipulation of resource supply and biotic impact (e.g., fertilization, annual weeding, no weeding, and functional group removal) at the subplot level. Biomass harvest by plant functional groups (forbs, grasses, climbers, and woody seedlings) on randomly located quadrates within each subplot can serve as a proxy for overall herb‐layer productivity and its functional group components. In combination with a vegetation survey, this approach allows assessing relationships between tree species richness and the presence of particular tree species in a plot with the richness, composition, and productivity of the herb layer. Such a combined approach reveals the extent to which these relationships change at different environmental settings when taking spatial heterogeneity at the site level into account.

### 2.2 Facets of tree diversity

#### 2.2.1 Leaf functional trait diversity

A primary goal of BEF research is to identify linkages between functional plant traits and ecosystem processes (de Bello et al., 2010; Díaz et al., 2007). In particular, the detection of key functional traits and their interrelationships and trade‐offs is of great importance to derive a mechanical understanding of ecosystem functioning. For example, the link between key functional leaf traits (e.g., nitrogen concentration and specific leaf area) and photosynthetic capacity as well as carbon capture has been well established in across‐site studies (Wright et al., 2004), but also occurs among subtropical forest tree species within sites. However, destructive sampling and time‐consuming analyses often limit sample size. Thus, it is desirable to assess plant traits including intraspecific trait variation in high spatial and temporal resolution by nondestructive and cost‐efficient high‐throughput methods. Near‐infrared reflectance spectroscopy (NIRS) has only recently been introduced to ecological research (e.g., Serbin, Singh, McNeil, Kingdon, & Townsend, 2014; Zuppinger‐Dingley, Flynn, Brandl, & Schmid, 2015), although it is a well‐established method for plant chemical analyses. As many leaf properties such as foliar C, N, phenolics, or leaf dry‐matter content show specific NIR reflectance spectra, target leaf traits can be easily assessed at different scales, from ground leaf powder to fresh leaves, entire tree canopies or forest ecosystems, once compound‐specific calibrations have been established (Couture et al., 2016; Foley et al., 1998). However, calibration requires a sufficiently high number of reference samples (approx. 200–300) with known trait information to yield reliable predictions for NIR spectrometry (Eichenberg et al., 2015).

Special attention has been given recently to field portable instruments which allow on‐site and nondestructive measurements, thereby making sample preparation and transport unnecessary (Galuszka, Migaszewski, & Namiesnik, 2015; Serbin et al., 2014). Portable spectrometers accelerate data collection and make it possible to consider intraspecific trait variation, for example, within‐canopy variation or temporal variation of leaf traits. An initial study conducted in the BEF‐China experiment on 4,892 leaves from 2,759 trees showed that important leaf traits (e.g., leaf dry‐matter content, specific leaf area, and C:N ratio) could be reliably predicted by portable field spectroscopy (Tobias Proß, unpublished data). It has been shown that quality of prediction differs among leaf traits because the high NIR absorption of water can reduce spectral information of other target components in fresh leaf samples. However, high measuring resolution outperforms potential drawbacks such as lower data quality and calibration efforts (Galuszka et al., 2015), which makes portable field spectroscopy an effective high‐throughput method for assessing leaf traits in large tree diversity experiments.

#### 2.2.2 Genetic diversity

Genetic diversity, especially heritable genetic variation in plant traits and in trait plasticity, causes large variation in plant performance (Frankham, 1999; Zeng, Durka, Welk, & Fischer, 2017) and represents the raw material for future adaptive evolution. Genetic diversity should therefore be considered as an additional facet of diversity that can influence plant performance in biodiversity experiments (Booth & Grime, 2003; Hahn et al., 2017; Schmid, 1994; Zeng, Durka, & Fischer, 2017). Genetic variation is generally found in studies on variation between plants from different genetic entities, such as provenances, populations, or maternal seed families. Moreover, different genotypes often respond differently to environmental variation resulting in genotype–environment interactions (Stearns, 1992).

In experimental analyses of biodiversity–functioning relationships, two issues should be considered. First, controlling for variation among genetic entities of the planted material (e.g., seed families and provenances) very much increases the resolution and statistical power for finding variation at the species level. Thus, in tree diversity experiments, the genetic identity of planted trees should be considered, for example using maternal seed families. Seeds of a maternal plant representing a seed family need to be collected, and seedlings need to be raised with recorded seed family identity. Seed family identity then needs to be considered during the experimental set‐up to control genetic variation, for example, by planting representatives of an equal number of seed families in all plots. Moreover, genetic variation can be manipulated using different numbers of seed families, for example, in order to assess the relative role of genetic variation at the inter‐ and intraspecific level (Hahn et al., 2017; Zeng, Durka, & Fischer, 2017).

Second, the omnipresence of genotype‐by‐environment interactions suggests that different genotypes may respond differently to experimental environments. Thus, members of seed families planted into experimental plots of different species diversity may serve as phytometer plants (Gibson, 2002; Mwangi et al., 2007) for diversity effects. Such phytometer plants offer the advantage that they can be planted into all experimental plots.

In the BEF‐China experiment, trees of known seed family were used (1) as matrix species in the main experiment, where for 12 species (~58.000 planting positions), seed family identity was recorded; (2) in a factorial species diversity × genetic diversity experiment, where genetic variation was manipulated using different numbers of seed families (Hahn et al., 2017); (3) as an additional phytometer by planting *Machilus thunbergii* seed families into each plot of the experiment.

Using seed families as matrix species or as phytometers allows to assess the heritability, that is, the amount of heritable genetic variation, in plant performance or plant traits using quantitative genetic methods and assuming a certain sibship coefficient between maternal seed families (e.g., ¼ for the case of half‐sib relations; Falconer, 1989; Lynch & Walsh, 1998; Zeng, Durka, Welk, et al., 2017). Moreover, genetic variation in phenotypic trait plasticity will become apparent, if seed families respond differently to differences between experimental treatments (Scheiner & Lyman, 1989). In conclusion, the use of multiple maternal seed families is a powerful experimental tool to increase the statistical power to detect variation at the species level, to quantify the heritability of plant traits and their plasticity, and to experimentally manipulate genetic variation.

### 2.3 Aboveground multitrophic interactions

#### 2.3.1 Herbivory

Herbivory directly affects resource allocation, trait expression, and plant growth (Agrawal, 2007; Coley & Barone, 1996; Viola et al., 2010). These factors all influence plant community composition, primary production, and nutrient cycling (Schmitz, 2008). Large‐scale herbivory assessments often trade‐off time efficiency and sampling accuracy, and quantification of leaf damage has become a standard method. Leaf damage is measured by either recording herbivory rates (increase in damage between two time points) or by measuring standing damage levels (i.e., one‐time measurements). For large‐scale BEF experiments, we recommend the latter, less time‐consuming method as a quick assessment tool (see also Johnson, Bertrand, & Turcotte, 2016). However, care needs to be taken as differences in leaf age can compromise comparisons among species (Poorter, van de Plassche, Willems, & Boot, 2004). We therefore recommend to use young (current season) leaves. If time of leaf flush differs substantially among tree species (which is not the case in BEF‐China; Schuldt et al., 2012), these differences need to be considered in assessment timing and data interpretation. Most studies visually estimate leaf damage, by either comparing total and damaged leaf area (Poorter et al., 2004) or using predefined damage classes (Schuldt, Bruelheide, et al., 2015; Sobek, Scherber, Steffan‐Dewenter, & Tscharntke, 2009; Unsicker et al., 2006; Vehviläinen, Koricheva, & Ruohomäki, 2007). For the BEF‐China project, predefined damage classes (0%, ≤5%, ≤25%, ≤50%, ≤75%, and >75%) have proven useful. Estimation accuracy has been assessed with digital scans of randomly collected leaves (Schuldt et al., 2012). For each tree, seven young, fully expanded leaves are screened on each of three randomly selected branches. With increasing tree height, branches are selected to represent upper, mid, and lower crown conditions. The sampling design follows the assessment of tree growth, comprising 6 × 6 individuals in monocultures and two‐species mixtures and up to 12 × 12 individuals in the more species‐rich plots. As the number of trees of a certain species per plot decreases with increasing tree diversity (because of constant planting density), an increase in the number of sampled trees per plot is necessary to allow for species‐level analysis at the tree level. Such analysis requires that all species are represented by a similar amount of tree individuals irrespective of the level of tree diversity (Bruelheide et al., 2014; Schmid, Baruffol, Wang, & Niklaus, 2017).

#### 2.3.2 Plant–fungal pathogens interactions

Parasitic interactions between plant hosts and fungal pathogens often cause a reduction in individual plant fitness by fungal consumption of photosynthetic products and negatively affect photosynthesis rates (Alves, Guimarães, Chaves, DaMatta, & Alfenas, 2011; Berger, Sinha, & Roitsch, 2007; Mitchell, 2003). The diversity and species composition of the plant host community affect fungal dispersal, infection, and infestation, mainly through negative density effects (Hantsch, Bien, et al., 2014; Hantsch, Braun, et al., 2014; Moore & Borer, 2012; Ostfeld & Keesing, 2012).

One advantage of a noninvasive rapid leaf damage assessment is the investigation of a high number of leaves and individuals of different plant species. For species comparability, we only use well‐formed leaves from the current year which are macroscopically screened for leaf damage caused by fungal spot and lesion symptoms, mildews, rusts, and sooty molds, respectively, at the end of the vegetation period. Similar to the herbivory assessment, total fungal damage is evaluated by damage classes (i.e., 0%, ≤5%, ≤25%, ≤50%, ≤75%, and >75%) on seven leaves randomly chosen from three different branches (representing different crown conditions), which were randomly selected per tree individual. The fungal damage assessment included (like other tree‐level measurements) an increasing number of tree individuals with increasing tree diversity to ensure a representative number of individuals per tree species per plot (i.e., 6 × 6 individuals in monocultures and two‐species mixtures, 9 × 9 individuals in four‐species mixtures, 12 × 12 individuals in eight‐, 16‐, and 24‐species mixtures).

In contrast to the more common microscopic in‐depth investigation of fungal pathogens (Hantsch, Braun, Scherer‐Lorenzen, & Bruelheide, 2013; Hantsch, Bien, et al., 2014) or identification of foliar fungi with molecular high‐throughput sequencing (Nguyen et al., 2017), fungal damage assessment needs not only less time allowing a higher sample size, but also works without specific expertise about fungal species.

#### 2.3.3 Trophobiosis

Tritrophic interactions between plants, sap‐sucking Hemiptera (e.g., aphids), and tending ants, so‐called trophobioses, are common in forests across climate zones (Ivens, von Beeren, Blüthgen, & Kronauer, 2016) and thus an ideal model system to quantify multitrophic interactions in forest BEF experiments.

We suggest and use in BEF‐China the following simple protocol for trophobiotic interactions that allows time‐efficient sampling of large numbers of trees (Staab, Blüthgen, & Klein, 2015). On each tree, at least 20 young leaves together with the attached branch sections are visually inspected for the occurrence of sucking Hemiptera and tending ants. If possible, surveys should be carried out monthly covering the main growing season. For Hemiptera and ant species that cannot be reliably identified in the field, voucher specimens are collected and stored in 70% ethanol for later identification. To ensure the sampling of a sufficiently large number of individuals of all tree species also in high‐diversity plots, we suggest increasing the number of sampled tree individuals with the tree diversity level of a given plot (see *Herbivory*). The data can be analyzed for the effect of tree species identity and tree species diversity. The R‐package “bipartite” offers all tools for ecological network analyses (Dormann, Fründ, Blüthgen, & Gruber, 2009). From our experience, network‐level specialization H_2_′ (Blüthgen, Menzel, & Blüthgen, 2006) and weighted generality G_qw_ (Bersier, Banašek‐Richter, & Cattin, 2002) are particularly useful to analyze the specificity and generality of plant–Hemiptera and Hemiptera–ant associations in response to tree diversity.

Besides simple and efficient sampling and data evaluation, a great advantage of trophobioses is that two fundamentally different forms of trophic interactions, consumption and mutualism (Thébault & Fontaine, 2010), can be studied simultaneously. If aphids are attacked by parasitoids, another trophic interaction can be added to the study system allowing an assessment of the ecosystem function parasitism (e.g., Gagic et al., 2011).

### 2.4 Belowground microbial interactions

#### 2.4.1 Microbial diversity

Soil microbes are crucial components of terrestrial ecosystems. They deliver key ecosystem functions and influence important ecosystem processes, including nutrient cycling and nutrient acquisition (Bardgett & van der Putten, 2014). Recent advances in next‐generation sequencing (NGS) techniques coupled with meta‐barcoding approaches and the associated bioinformatics and statistical analysis tools enabled microbial ecologists to work in large‐scale tree diversity experiments to shed light on the poorly understood role of microbial diversity on BEF relationships in forest ecosystems.

Although the advance in NGS and the possibility to analyze a large number of samples have led to large‐scale and integrated biodiversity studies at the global scale (Shoemaker, Locey, & Lennon, 2017), standardized soil sampling, storage, and transportation across continents still are a challenge. Accordingly, we developed a soil sampling, freeze‐drying, and preservation protocol that guarantees transportation of soil samples without nucleic acid degradation between laboratories across continents (Weißbecker, Buscot, & Wubet, 2017). The soil microbial nucleic acid extraction protocols have been optimized to a high‐throughput protocol, and the classical PCR‐based microbial diversity analysis protocols using microbial rDNA‐based barcodes (e.g., 16S for bacteria and ITS for fungi) have been adapted to meta‐barcoding protocols using NGS platforms (Lentendu et al., 2014; Wu et al., 2013).

Another crucial point is the sampling strategy. Soils are anything but a homogenous compartment, and even within each horizon, they are a complex patchwork of microhabitats with variable levels of resources and very specific communities. In BEF experiments, a crucial decision is whether to sample the roots and rhizosphere of each plant species used in the design or to sample the bulk soil. The rhizosphere has a selective filtering effect differing between plant species, while the bulk soil may better reflect the general effect of a plant biodiversity level on the whole microbial community. Even for mycorrhizal fungi directly linked to plant roots, it was shown in grassland studies that analyzing bulk soil better captures biodiversity than focusing on roots (Hempel, Renker, & Buscot, 2007). In addition, preliminary analyses in BEF‐China found not only the highest soil microbial biomass and activities in the uppermost horizon under the plant litter, but also that this was the most reactive soil layer to variations in the biodiversity and age structure of the trees and understory (Wu et al., 2012). Based on our experience, we recommend that broad analyses of soil microbial communities in BEF experiments should be based on multiple samples from the upper soil layer at equal distance from neighbor plants. These samples can be pooled into a composite sample from which the DNA is extracted and analyzed (Wu et al., 2013).

Integrating the microbial species (operational taxonomic units—OTU) abundance matrices with other co‐occurring organisms and environmental variables and using ecological statistical analysis tools enabled us to assess the significance of soil microbes on inter‐ and intrakingdom interaction networks, multitrophic interactions, forest ecosystem functions, and multifunctionality.

#### 2.4.2 Microbial biomass and activity

The effects of tree species diversity on soil microbial community structure and activity remain poorly understood, despite the important role of soil microorganisms for ecosystem functioning (Naeem et al., 2000; Zak, Holmes, White, Peacock, & Tilman, 2003).

Phospholipid fatty acid analysis (PLFA) has been validated as a valuable approach of investigating soil microbial community composition and viable microbial biomass (Bartelt‐Ryser, Joshi, Schmid, Brandl, & Balser, 2005; Frostegård & Bååth, 1996; Frostegård, Tunlid, & Bååth, 2011; Pei et al., 2016; Vestal & White, 1989). Recently, a high‐throughput method of lipid extraction and analysis has been developed, which allows for lipid profiling for large ecosystem studies (Gutknecht, Field, & Balser, 2012; Oates et al., 2017). In this method, the initial soil chloroform extraction is carried out in the standard procedure (“modified” Bligh and Dyer (1959) extraction) and then followed by the FAME procedure of saponification, acid methylation, and extraction (Schutter & Dick, 2000). This high‐throughput method retains the sensitivity of traditional PLFA methods, but allows for much more rapid analysis of a large number of samples, for example enabling us to demonstrate how tree species identity and growth traits interact with soil characteristics across a large number of tree species to shape soil microbial growth (Pei et al., 2016). Another benefit of PLFA analysis is that the bacterial, fungal, or total microbial carbon pools can be calculated, for comparison with other measures of productivity and carbon cycling (Schmidt, Schulz, Michalzik, Buscot, & Gutknecht, 2015).

Besides, microbial species composition it is also important to understand how forest diversity alters microbial functional processes. To do this, we used a modification of the ^15^N pool dilution approach (Stange, Spott, Apelt, & Russow, 2007) based on traditional methods (Booth, Stark, & Rastetter, 2005; Hart, Stark, Davidson, & Firestone, 1994). The ^15^N isotope pool dilution approach can quantify gross rates of N mineralization, nitrification, and microbial immobilization. The limitation of this method is that it necessitates the usage of fresh soil and the usually laborious process of precipitating salt extractions for isotopic analysis (Hart et al., 1994). For analysis of extractions, we used a new spin mass system to analyze ^15^NO_3_ and ^15^NH_4_ directly from liquid samples (Stange et al., 2007), nearly halving the processing effort.

In addition to microbial nitrogen processing rates, soil microbial decomposition potential, measured through extracellular enzyme activities, is an important functional trait of microbial communities. For example, we are using this method to establish how forest and litter diversity alter decomposition through changes in soil microbial activities (Z. Pei, unpublished data). We examine extracellular enzyme activity according to the method described by Saiya‐Cork, Sinsabaugh, and Zak (2002) and recently modified by DeForest (2009) and German et al. (2011). Due to the small‐scale analysis in 96‐well plates and the use of multiwell plate‐reader technology, rapid processing of a large number of samples is feasible. With these methods, we are able to process several hundred soil samples per campaign in order to capture both individual‐species and plot‐level changes in microbial growth and activity (Pei et al., 2016).

### 2.5 Nutrient cycling

#### 2.5.1 Leaf litter decomposition

Decomposition of organic matter is a highly integrative process in ecosystem biogeochemistry, which replenishes the pool of plant available nutrients, and releases photosynthetically fixed carbon back to the atmosphere (Berg & McClaugherty, 2008). Species diversity effects on litter mass loss and nutrient release have been reported at the level of plants and detritivores (Gessner et al., 2010; Hättenschwiler, Tiunov, & Scheu, 2005).

Litterbags filled with a standard litter substrate are commonly used to study diversity effects that act via changes in the microenvironment induced by tree diversity or species composition. If leaf litter of tree species planted in the experiment serves as standard substrate, the home‐field advantage should be considered as potential bias because decomposition of plant litter might be faster on plots where the same species is planted (Ayres et al., 2009; Freschet, Aerts, & Cornelissen, 2012). To increase comparability across decomposition studies, common tea bags have recently been suggested as standardized litterbags and fast assessment tool (Keuskamp, Dingemans, Lehtinen, Sarneel, & Hefting, 2013). This low‐cost and time‐efficient approach allows a large sample size and can thus help to assess tree diversity effects on decomposition dynamics by combining data from experiments across the globe. However, the standard material used (green tea, rooibos tea) is absent from the studied ecosystem, hence it will be difficult to infer the multitude of mechanisms by which tree diversity may influence litter decomposition. Magnitude and direction of tree diversity effects can also differ among litter substrates. Thus, to account for possible species identity effects, plant litter with contrasting litter quality should be employed together as standard litter substrates (Seidelmann et al., 2016). As with any other standard material used (e.g., wheat straw, cotton strips, and standard litter of one species), only tree diversity effects that act via changes in the microenvironment can be assessed, but not any effects that act via the quality of litter present in the ecosystem (Scherer‐Lorenzen, 2008). Thus, in addition, we suggest to measure community‐specific litter decomposition in the corresponding plots to account for the combined effect of microenvironment and litter quality. Finally, to isolate the effects of litter quality, single‐species litterbags can be incubated in a common plot providing a homogeneous environment (Trogisch, He, Hector, & Scherer‐Lorenzen, 2016).

In large tree diversity experiments, a high number of litterbags are required to include as many plots as possible. For example, we used a total of 3,618 bags which were exposed on 402 subplots in the BEF‐China experiment with bags retrieved after 2, 6, and 11 months (Seidelmann et al., 2016). Thus, preparation time of litterbags including collection of site‐specific plant litter should not be underestimated.

The mesh should be UV‐resistant in case bags are not buried but are exposed to high solar radiation. The chosen mesh size strongly controls the access for decomposer organisms, and a trade‐off between small mesh size (excluding macrofauna, but minimizing the loss of litter fragments) and large mesh size (allowing access of most organisms, but increasing the risk of losing litter fragments) exists (Bradford, Tordoff, Eggers, Jones, & Newington, 2002; Prescott, 2005). To cope with this trade‐off, litter bags with a micromesh (e.g., 50 μm) at the bottom part of the bag that has contact to the soil, and larger macromesh (e.g., 5 mm) at the top of the bag can be used (Harmon, Nadelhoffer, & Blair, 1999).

#### 2.5.2 Deadwood decomposition

Deadwood is a key driver of ecosystem functioning in forests (Cornwell et al., 2009; Harmon et al., 1986; Wirth, 2009) and one of the most important components of forest ecosystem biodiversity, carbon and nutrient cycling, energy flows, and soil‐forming processes (Harmon et al., 1986; Laiho & Prescott, 1999; Lindahl, Taylor, & Finlay, 2002). On the one hand, care must be taken when choosing the size of wood samples with respect to the scope of individual studies. Smaller pieces allow a larger sample size with a feasible amount of labor and space requirements in the field. On the other hand, larger pieces can carry a higher diversity of decomposers due to the fact that especially larger decomposer species (e.g., cerambycid beetles) prefer larger wood pieces for development. We chose standard‐sized stem wood of 25 ± 1 cm length and 8 ± 2 cm diameter (Eichenberg et al., 2017). The influence of certain deadwood decomposer organisms such as termites and other invertebrates is studied using different mesh sizes in a litterbag approach (Eichenberg et al., 2017). This allows a fast assessment of abiotic controls on wood decomposition in relation to invertebrate plus fungal‐ and microbial‐mediated versus exclusively fungal‐ and microbial‐mediated decay. Litterbags also ensure that no samples or fragments of samples are lost in steep terrain over the course of the experiment. In our case, replicated bags with wood pieces were retrieved one and 3 years after deposition. Similar to the tea bag index for leaf litter (Keuskamp et al., 2013), a common protocol defining standard wood substrates (i.e., ice cream sticks from birch wood and chopsticks) would greatly expand the comparability of wood decomposition rates for better global predictions.

#### 2.5.3 Soil fertility and C storage

Soil fertility is an important covariate in the analysis of effects of tree species richness on ecosystem functioning. Large forest BEF experiments, in particular those in geomorphologically heterogeneous landscapes, have inherently a considerable spatial variation in many attributes that also influence soil nutrient availability and fertility (e.g., Scholten et al., 2017).

Quantifying abiotic site conditions including soil nutrients is therefore critical for interpreting biodiversity effects on forest stand performance. Moreover, regular inventories of sensitive soil nutrient pools (e.g., content of available and N and P) in 5‐year intervals may yield important insights into how tree species richness and composition modify soils during stand development. Tracking these plant‐induced temporal changes in soil properties (see ecoscape approach above) permits the identification of forest compositions promoting nutrient cycling and nutrient use efficiency (Richards, Forrester, Bauhus, & Scherer‐Lorenzen, 2010) and also the quantification of soil C accumulation—an important ecosystem service (Díaz, Hector, & Wardle, 2009).

In the BEF‐China experiment, initial soil conditions have been thoroughly mapped before forest establishment (Scholten et al., 2017). Systematic soil sampling included taking nine soil cores in each plot to a depth of 50 cm which were pooled per plot and soil layer (0–5, 5–10, 10–20, 20–30, and 30–50 cm). Soil fertility has been characterized by measuring total soil carbon, nitrogen, soil pH, cation‐exchange capacity, exchangeable cations, and base saturation. Many of these properties can also be determined with sufficient accuracy through near‐infrared spectroscopy (NIRS) and mid‐infrared spectroscopy (MIRS), once calibrated for the particular soil property, to facilitate inexpensive analyses and rapid assessment of large numbers of samples in subsequent inventories (e.g., Chen, Dong, Li, & Wang, 2017; Ludwig, Khanna, Bauhus, & Hopmans, 2002). Where information is to be gathered for entire soil profiles, the soils still need to be sampled conventionally (e.g., with corers) before soil samples can be analyzed with these indirect methods. For soils of the BEF‐China experiment, NIRS models were developed to replace the onerous Hedley method employing a wet‐chemical process of determining fractions of soil P corresponding with different plant availability through sequential extraction of samples (Niederberger et al., 2015). The potential of NIRS to save time and costs is particularly high for soil properties that cannot be determined through a single chemical analysis but require incubation approaches or repeated extractions, for example, nitrogen and carbon mineralization rates (e.g., Ludwig et al., 2002). In the context of BEF experiments, the approach may also be very interesting to trace the species origin of soil organic matter to disentangle the influence of tree diversity on soil carbon stocks (e.g., Dobarco, van Miegroet, Gruselle, & Bauhus, 2014).

### 2.6 Soil erosion control

Large tree diversity experiments require a broad range of combined techniques to assess soil erosion processes. Measurements address the kinetic energy of raindrops (splash cups), runoff and sediment discharge (runoff plots), and long‐term monitoring (erosion sticks).

Splash cups consist of a plastic flask attached to a carrier system, filled with a unit sand of 125–200 μm particle size (Scholten, Geißler, Goc, Kühn, & Wiegand, 2011). The sand loss calculated from the amount of sand remaining after exposition of the cup to rainfall is converted to kinetic energy using a linear calibration function derived from laser precipitation monitor measurements (Lanzinger, Theel, & Windolph, 2006). Splash cups are light, reliable and allow a high number of replications on different positions under a tree. Results permit detecting differences in kinetic energy between different tree species and diversity levels (Geißler et al., 2013; Goebes, Bruelheide, et al., 2015; Goebes, Seitz, et al., 2015).

Surface runoff and sediment discharge are observed using microscale runoff plots (ROPs) sized 0.16 m^2^ (0.4 m × 0.4 m) and bordered by stainless steel panels in which soil surface cover (e.g., by stones or biological soil crusts) is recorded photogrammetrically (Seitz et al., 2016). ROPs can be equipped with pitfall traps to implement a soil fauna treatment (Seitz et al., 2015). Runoff is collected in 20‐L containers connected to covered triangular gutters. Both sediment discharge and runoff are analyzed for C, N, and P contents. The small ROP size allows investigating interrill erosion precisely as other processes like rill erosion do not occur on such short flow distances (Agassi & Bradford, 1999) and those small ROPs are particularly appropriate to compare different diversity treatments (Wainwright, Parsons, & Abrahams, 2000). A further advantage is the possibility to use a high number of randomized replications at a time (220 ROPs in BEF‐China), which is an important precaution in the design of ROP measurements (cf. Hudson, 1993).

Long‐term monitoring of soil erosion characteristics on over 500 plots in the BEF‐China experiment requires a reliable and cost‐efficient technique (Shi, Wen, Zhang, & Yan, 2011). Erosion sticks, 1‐m long UV‐resistant PVC rods, are pushed into the soil at nine positions in each plot. Approximately 4,500 erosion sticks have been installed in the BEF‐China experiment, and the length of the sticks above the soil surface is measured once per year.

## 3 DISCUSSION

**Figure 3:**
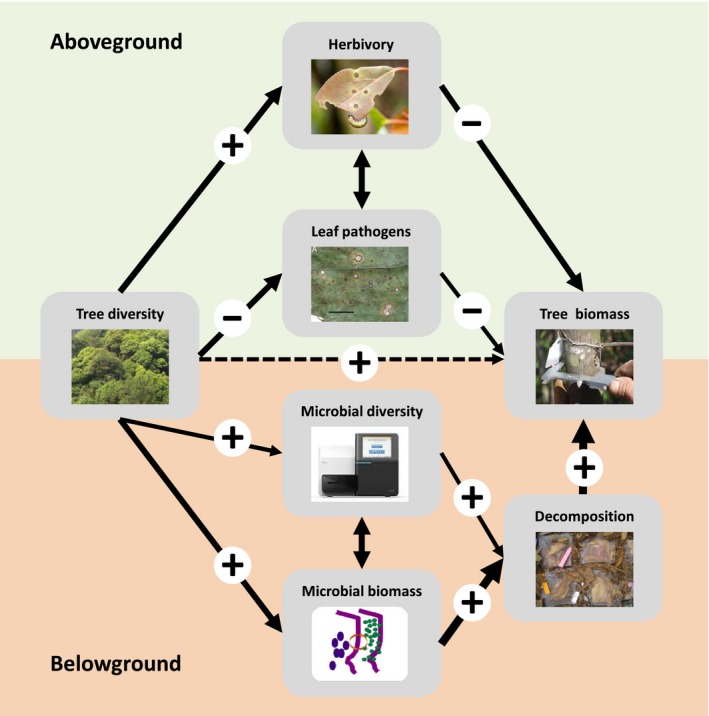
Identifying the links and underlying mechanisms between tree diversity and key ecosystem functions requires the coordinated assessment of forest multifunctionality across trophic levels and ecosystem subsystems. For example, consistent datasets of relevant ecosystem functions are needed to analyze the effect of tree diversity on tree biomass using structural equation modeling. Shown is a simplified conceptual structural equation model which links aboveground (herbivory, leaf pathogen infestation) and soil‐related processes (soil microbial biomass and diversity, decomposition of leaves and roots and deadwood decomposition) affecting tree biomass. Solid and dashed arrows show hypothetical significant and nonsignificant positive or negative effects, respectively. Increasing arrow width specifies hypothetical strength of causal relationship between variables. Positive and negative relationships are indicated by “+” and “−” signs, respectively

Based on methods currently applied in one of the world's largest tree diversity experiments, we highlighted how methods can be combined to simultaneously address multiple ecosystem functions and consequently maximize synergy in forest biodiversity research. By implementing harmonized methods, scientific knowledge gain can be optimized while simultaneously using the specific expertise of involved research teams efficiently. Only if consistent datasets for essential ecosystem functions can be amalgamated within and across tree diversity experiments, progress in BEF research can be achieved. For example, understanding how herbivory and leaf pathogens are influenced by tree diversity can provide deeper insights into the importance of multitrophic interactions for tree biomass (Schuldt, Bruelheide, et al., 2015) (Figure 3). Similarly, decomposition dynamics along tree diversity gradients can only be explained when we know how tree diversity affects microbial activity and the diversity and composition of decomposer communities. Ultimately, the combination of above‐ and belowground processes can help to identify direct and indirect drivers of vital ecosystem functions such as biomass production across ecosystem subsystems (Figure 3).

In order to fully explore the potentials of tree diversity studies that aim to quantify effects on multifunctionality, an “all‐measurements‐on‐all‐plots philosophy” should be adopted, despite the large number of plots (Baeten et al., 2013). This strategy might, however, restrict the choice of methods, as often such high‐throughput methods can rather be seen as “proxies” for the ecosystem function of interest, because more sophisticated or detailed measurements are too time‐consuming or expensive. Based on the knowledge we have gained from the BEF‐China and other forest BEF experiments, we propose the following guidelines for present and future tree diversity experiments.

### 3.1 Maximizing data density

Given the high number of tree individuals planted in tree diversity experiments, often only a subset of individuals can be measured in each plot. In particular, this is true for ecosystem functions like tree growth that require annual or even more frequent measurements of individual trees. Different methods therefore have a different range in terms of their spatial and temporal resolution. Whereas some methods are easily applicable to a relatively large subset of tree individuals per plot (e.g., portable spectrometers), others are restricted to only a few individuals due to high work intensity and time constraints (e.g., minirhizotrons). Thus, methods with a high sample size should always comprise those tree individuals or plot areas that are assessed by methods with a smaller range. The goal should be to maximize data density, that is, the number of measured variables, for a given subset of tree individuals in each plot. For example, in BEF‐China, most measurement activities focus on the central 4 × 4 to 12 × 12 tree individuals in each plot. This means that for a certain subset of individuals, data on productivity, litter production, tree growth, microbial biomass, the plant microbiome, herbivory, or foliar fungal pathogen infestation are available and can be correlated at the tree level.

Furthermore, the combination of several rapid nondestructive methods allows measurements even on the same branches or leaves (e.g., leaf trait assessment using NIRS combined with herbivory survey). Ideally, aboveground and belowground methods should focus on the same tree individuals to increase data density across subsystems. In monocultures and low‐diversity mixtures, the number of measured tree individuals can be reduced because of the high number of replicates (see above). The quantification of multifunctional responses at individual tree level to neighborhood‐ or plot‐level implies that variables must be measured on the same tree individuals, which requires well‐coordinated and time‐adjusted measurement campaigns among involved research teams. Sampling effort can be considerably reduced if collected samples are shared among project partners. For example, subsamples of soil cores taken for nutrient analysis can be used for investigating soil microbial communities (Pei et al., 2016). Similar, different aspects such as nutrient cycling and microbial community composition can be effectively studied in joint decomposition experiments when taking a shared sampling strategy into account (Pei et al., 2017; Purahong et al., 2017).

### 3.2 Applied methods should cover relevant scales

Tree diversity experiments with their large spatial extent are usually established with a long‐term view on measurement activities and data acquisition. Thus, chosen methods should consider relevant spatial and temporal scales. The relationship between biodiversity and ecosystem functioning has been predominantly analyzed at the level of the community or plot, thereby neglecting the scale dependency of diversity effects (Chisholm et al., 2013; Schuldt, Wubet, et al., 2015). However, biotic interactions which determine the strength of biodiversity effects occur at the tree individual scale (Potvin & Dutilleul, 2009) and can be influenced by intraspecific (genotypic) trait variation (Johnson, Lajeunesse, & Agrawal, 2006) as well as the direct tree neighborhood (Barbosa et al., 2009). In BEF experiments, fully mapped and geo‐referenced tree positions allow testing for neighborhood relationships at different scales. Thus, it is not necessary to decide beforehand which scale is appropriate, but instead it is best to apply a spectrum of methods that can capture local neighborhood interactions up to stand‐level dynamics. For example, upscaling water use from individual trees to neighborhoods to plot (community) level needs data on xylem flow rates measured on individual trees and reliable estimates of sapwood area at plot level (Kunert, Schwendenmann, Potvin, & Hölscher, 2012).

It is clear that each method tends to focus either on individual trees (e.g., herbivory assessment) or on the plot (community) level (e.g., litterbags, erosion sticks), which might require a trade‐off between generality and precision for the large number of trees to be measured. Thus, methods should be ideally combined in a way that they bridge precision and generality. This critical trade‐off between precision and generality should be methodologically addressed in order to allow reliable upscaling of the BEF relationship to relevant scales for ecosystem management.

### 3.3 Consistency in method selection in time and space

It is necessary to adapt methods to tree size and forest development stage. For some ecosystem functions, this sometimes requires an inevitable change in methods. For example, while tree canopy measurements are easily carried out in the first years after planting, this is usually not the case anymore after trees have reached a certain height. Leaf demographic assessments using marked leaf cohorts are not practical anymore after trees have reached a certain height and are replaced by collecting leaf and fine twig litter fall in litter traps. Similarly, sampling for herbivory or plant pathogen assessment needs to be adapted to increasing tree height by considering lower, mid, and upper canopy layers. However, newly introduced methods or adapted sampling designs should always be consistent, that is, calibrated and validated compared to previously used approaches. Consistency in applied methods should be promoted to ensure adequate data analysis of long‐time series and to reduce ecological uncertainty (Schimel & Keller, 2015). This is especially important given that biodiversity effects may develop and become stronger over time. For example, microbial adaptation to certain tree species over time can alter aboveground–belowground interactions and could influence or reinforce biodiversity effects (Mangan et al., 2010). However, consistency of time series measurements may be compromised by fluctuation in the composition of research teams, available funding, or adjustment of research questions during the lifetime of the experimental platform. To ensure that knowledge on respective methods is not lost with time, collected datasets should be linked to respective technical protocols in the platform's database. Publishing methods in novel formats such as scientific video journals could further promote reproducibility and consistency of measurements (Kröber, Plath, Heklau, & Bruelheide, 2015). On a wider level, reducing ecological uncertainty by application of consistent and standardized methods across globally distributed experimental forest sites would improve the evaluation of general tree diversity effects (Fraser et al., 2012). In the long run, we think that a central web platform that compiles innovative methods and provides detailed protocols would largely promote data harmonization in cross‐site experimental studies on forest multifunctionality.

Moreover, large BEF experiments offer an ideal test platform for introducing new emerging methods in forest diversity research. For instance, drone‐based remote sensing is currently a rapidly developing technology (Tang & Shao, 2015). Drone remote sensing has been successfully tested for example in forest inventories and to estimate tree canopy height and canopy closure (Getzin, Wiegand, & Schöning, 2012; Torresan et al., 2017). As tree positions in BEF experiments are fully mapped, remote sensing data can be easily related to ground‐based measurements such as of DBH or LAI. In this way, the overlap with already well‐established approaches not only ensures better calibration and consistency but also promotes the establishment of new technologies.

### 3.4 Promoting rapid assessment of biodiversity and ecosystem functions

The scale of sampling in large tree diversity experiments necessitates rapid, standardized, and cost‐effective assessment of biodiversity. These have been successfully developed for taxa such as arthropods (Obrist & Duelli, 2010; Oliver & Beattie, 1996; Yu et al., 2012), and meta‐genomic methods are used for rapid multitaxa assessment of microbial and fungal diversity (Cannon, 1997; Gao et al., 2015). The bottleneck of the “taxonomic imperative” can be addressed with DNA‐based methods, particularly those based on NGS of pooled communities (Yu et al., 2012). These use quantified criteria for delineation of species diversity (Pons et al., 2006) and assignment of taxonomic names (Hebert, Ratnasingham, & deWaard, 2003), allow a greatly increased throughput (Ji et al., 2013), and are amenable to digital storage and meta‐analysis in a web‐based framework (Ratnasingham & Hebert, 2013). DNA barcoding can be adapted to take advantage of greater information content of multigene and PCR‐free sequence data (Chesters, Zheng, Zhu, & Yu, 2015). Additionally, wiki‐based descriptions allow for integration with morphological taxonomy without imposing excessive time constraints (Riedel, Sagata, Suhardjono, Tanzler, & Balke, 2013).

With respect to plant functional diversity, morphological, and biochemical leaf traits that are known to be important for driving ecosystem functions can be quickly assessed by portable NIRS in the field, once calibration is established (see above). With its high sample throughput, NIRS makes it possible to study, for example, seasonal dynamics of leaf nutrients, which can offer new insights into trait variation at much finer temporal and spatial scales. NIRS can also help to resolve species composition in fine‐root mixtures (Lei & Bauhus, 2010) and to determine some soil properties such as available P, which are otherwise only quantifiable with onerous laboratory methods (Niederberger et al., 2015). In this way, high spatial and temporal resolution of trait measurements can be achieved which will improve trait‐based predictions of ecosystem functioning. The identification of easily measurable plant trait syndromes which reflect ecophysiological key functions could further strengthen this approach.

Besides rapid assessment of biodiversity there is a clear need to develop easy‐to‐use and quick methods for the measurement of key ecosystem functions. A standardized rapid ecosystem function assessment (REFA) has been recently suggested and conceptualized by Meyer et al. (2015). Low‐tech, easy‐to‐use, repeatable, and cost‐efficient measurements allow the harmonized assessment of ecosystem functions (e.g., biogeochemical cycles, tree productivity, or consumer–plant interactions) across a large number of plots and experimental sites. This approach is especially beneficial in a multifunctional context as the number of ecosystem functions considered in an experiment can be increased. Furthermore, in contrast to more traditional approaches, functions can be studied at the same spatial resolution, preferably on all plots or levels of tree diversity, due to reduced measurement effort. In this way, inherent interrelationships in multitrophic networks (Staab et al., 2015) or across below‐ and aboveground subsystems could be more adequately considered in BEF research. However, the measurement of ecosystem functions in structurally complex forest systems imposes special requirements in terms of spatial and temporal scale. This means that REFA methods and sampling designs need to be specifically adapted or developed for assessing forest multifunctionality. In this respect, our compilation of methods could serve as a first contribution for the development of a REFA framework for forests.

## 4 OUTLOOK

The majority of previous studies in forest BEF research have focused on single ecosystem functions, thereby neglecting inherent feedback mechanisms, essential connections between above‐ and belowground subsystems, and important trophic relationships. However, knowledge of these interdependencies among multiple functions is crucial to understand and predict the responses of forest ecosystems to species loss. Considerable progress in forest BEF experiments can be promoted by applying harmonized methodical approaches to comprehensively assess forest multifunctionality. Method selection should therefore be guided by major principles such as consistent application of methods across spatial and temporal scales, maximizing data density and rapid assessment strategies to increase the number of replicates. Another important issue is to ensure data comparability across tree diversity experiments for the growing number of synthesis initiatives. Ideally, this requires space‐ and time‐aligned measurement campaigns and common agreement on standardized protocols. Current methods need to be adapted to account for the specific requirements of structurally complex and long‐lived forest ecosystems. New innovative approaches such as the identification of easy‐to‐measure indicators for ecosystem functioning or other rapid assessment strategies have to be developed. With these challenges ahead, we hope that our outline of key methods currently applied in one of the largest tree diversity experiments will help to promote synergy and comprehensive assessment of multifunctionality in forest biodiversity research.

## AUTHOR CONTRIBUTIONS

HB, BS, and KPM conceived the initial ideas; ST, AS, and HB designed the paper and led the writing of the manuscript. All authors (ST, AS, JB, JAB, SB, FB, NC, DC, WD, DE, AE, MF, CG, MG, PG, JG, CH, SH, WH, JSH, AH, LH, YH, AMK, PK, MK, KL, YL, YJL, PN, ZP, KAP, RP, TP, MSL, KS, TS, SS, ZS, MS, GvO, ChW, EW, CW, TW, BY, XY, CDZ, BS, KPM, and HB) wrote individual chapters, contributed critically to the drafts, and gave final approval for publication.
